# The natriuretic peptide blind spot in obesity-related heart failure with preserved ejection fraction

**DOI:** 10.1097/MS9.0000000000005620

**Published:** 2026-09-10

**Authors:** Joshua Cho Mbony, Achu Fonkem

**Affiliations:** a Department of Internal Medicine, Presbyterian Health Complex, Yaoundé, Cameroon; b Department of Internal Medicine, Ministry of Public Health, Yaoundé, Cameroon; c Department of Internal Medicine, Jamot Hospital of Yaoundé, Yaoundé, Cameroon

**Keywords:** diagnostic testing, exercise hemodynamics, HFpEF, natriuretic peptide, obesity

## Abstract

Heart failure with preserved ejection fraction (HFpEF) is a heterogeneous syndrome in which obesity can influence both disease expression and diagnostic test performance. This editorial examines the clinical implications of low or apparently normal natriuretic peptide concentrations in obesity-related HFpEF. Published clinical and hemodynamic evidence indicates that some affected patients demonstrate pathological elevations in cardiac filling pressures, particularly during exercise, despite natriuretic peptide concentrations below conventional diagnostic thresholds. Although these patients may have less advanced structural disease and lower event rates than those with markedly elevated concentrations, they can still experience substantial functional limitation and worse outcomes than hemodynamically normal individuals. Reliance on fixed biomarker thresholds may therefore delay diagnosis and contribute to underrepresentation of this phenotype in clinical trials. Natriuretic peptides should remain central to HFpEF evaluation but should be interpreted in conjunction with the clinical phenotype, echocardiographic findings, validated probability scores, and selective exercise-based testing.

## Introduction

Heart failure with preserved ejection fraction (HFpEF) is a heterogeneous clinical syndrome characterized by symptoms and signs of heart failure, preserved left ventricular ejection fraction, and evidence of elevated cardiac filling pressures at rest or during exercise. Obesity is a common comorbidity that may contribute directly to HFpEF through plasma volume expansion, systemic inflammation, metabolic dysfunction, ventricular interaction, pericardial restraint, and impaired peripheral oxygen utilization. Patients with obesity-related HFpEF frequently present with exertional dyspnea and functional limitation despite only modest abnormalities on resting evaluation[1]. Recognition of this phenotype has become increasingly important, as sodium-glucose cotransporter 2 inhibitors and obesity-directed therapies have expanded the available treatment options. Accurate diagnosis is, therefore, not merely an exercise in disease classification, but an important step toward identifying patients who may benefit from targeted management.

B-type natriuretic peptide (BNP) and N-terminal pro-B-type natriuretic peptide (NT-proBNP) remain fundamental components of heart failure assessment because they provide valuable diagnostic and prognostic information related to myocardial stress and hemodynamic burden. Their interpretation in obesity, however, requires particular care. Circulating concentrations are generally lower in individuals with obesity, and some patients with hemodynamically confirmed HFpEF have values below conventional diagnostic thresholds. Resting echocardiographic indices may also be non-diagnostic when abnormal filling pressures emerge predominantly during exercise[2]. Consequently, an apparently normal natriuretic peptide concentration may reduce the estimated probability of HFpEF without reliably excluding it in a patient with a compelling clinical phenotype. This editorial examines the biological and clinical basis of this diagnostic blind spot, clarifies the appropriate role of natriuretic peptides in obesity-related HFpEF, and proposes a phenotype-aware approach for patients whose symptoms and clinical profiles remain suggestive despite reassuring biomarker results.

## The obesity-related HFpEF phenotype

Obesity-related HFpEF is not simply conventional HFpEF occurring in a patient with an elevated body mass index (BMI). Excess adiposity may produce a recognizable hemodynamic and systemic phenotype involving plasma volume expansion, sodium retention, neurohormonal activation, systemic inflammation, insulin resistance, and impaired skeletal muscle oxygen utilization. Visceral and epicardial adipose tissue may also promote ventricular interaction and external cardiac constraint. Collectively, these abnormalities can elevate cardiac filling pressures, particularly during exertion, despite relatively modest findings at rest. BMI does not distinguish between fat distribution and lean mass and may therefore incompletely represent the hemodynamic burden associated with central or visceral adiposity[3].

In an invasive hemodynamic investigation, Obokata *et al*[4] found that patients with obesity-related HFpEF had greater plasma volume expansion, more pronounced biventricular remodeling, increased epicardial fat, greater ventricular interaction, and higher filling pressures than patients with HFpEF without obesity. They also demonstrated poorer exercise capacity and more pronounced hemodynamic impairment during exertion. These findings support obesity-related HFpEF as a pathophysiologically meaningful phenotype rather than obesity functioning solely as an accompanying risk factor. Nevertheless, recognition remains challenging because obesity itself can cause dyspnea, physical deconditioning, and reduced exercise tolerance. Objective evidence of cardiac dysfunction remains necessary, but the tests used to obtain that evidence must be interpreted with an understanding of how obesity affects cardiac structure, hemodynamics, and biomarker expression.

## Why natriuretic peptides may remain normal

BNP and NT-proBNP are released in response to myocardial wall stress and provide important diagnostic and prognostic information in heart failure. Their circulating concentrations, however, are influenced by factors beyond cardiac filling pressure. Obesity is consistently associated with lower natriuretic peptide concentrations, including among patients with established HFpEF. Several mechanisms may contribute. Concentric ventricular remodeling can limit chamber dilatation and reduce wall stress relative to intracavitary pressure. Epicardial fat, increased total heart volume, and pericardial restraint may further increase external cardiac constraint, allowing pulmonary capillary wedge pressure to rise without a proportionate increase in transmural distending pressure or natriuretic peptide release[3]. Enhanced clearance through natriuretic peptide clearance receptors in adipose tissue may contribute to lower BNP concentrations, but this mechanism cannot fully explain the parallel reduction in NT-proBNP. Reduced peptide synthesis or secretion, insulin resistance, and sex-related hormonal effects have also been proposed, although their relative contributions remain incompletely defined[3].

Clinical investigations demonstrate the significance of this biological effect. Verbrugge *et al*[5] identified 157 patients with hemodynamically confirmed HFpEF despite NT-proBNP concentrations below 125 ng/L among individuals undergoing invasive cardiopulmonary exercise testing for unexplained dyspnea. Although these patients had less advanced structural disease than those with elevated natriuretic peptide concentrations, they developed markedly elevated filling pressures during exercise and had a 2.7-fold higher adjusted risk of death or heart failure readmission than hemodynamically normal controls. Complementary evidence was provided by Kondo *et al*[6] in an analysis of 3480 participants with symptomatic heart failure and a left ventricular ejection fraction of at least 45% in I-PRESERVE. Of these participants, 808 (23.2%) had NT-proBNP concentrations below 125 pg/mL. Although their risk of cardiovascular death or heart failure hospitalization was lower than that of participants with higher concentrations, their reported health status was similarly impaired. These findings do not diminish the value of natriuretic peptides. Instead, they show that values below conventional thresholds may identify a lower-risk HFpEF phenotype without reliably establishing normal hemodynamics, particularly in patients with obesity and exertional symptoms.

## How the blind spot affects diagnosis

The principal diagnostic consequence of natriuretic peptide suppression is false reassurance. In ambulatory patients with obesity, exertional dyspnea may be attributed to body habitus, pulmonary disease, or deconditioning when BNP or NT-proBNP falls below a conventional threshold. Conversely, obesity can produce dyspnea without HFpEF, so neither symptoms nor biomarker results can independently establish or exclude the diagnosis. When a convincing cardiometabolic phenotype is accompanied by discordant test results, a low natriuretic peptide concentration should reduce the estimated probability of HFpEF without automatically terminating further evaluation.

Resting assessment creates an additional blind spot. Some patients maintain near-normal filling pressures at rest but develop a pathological rise during exercise, when symptoms occur. Echocardiographic indices, including the ratio of early mitral inflow velocity to early diastolic mitral annular velocity, may also underestimate congestion in obesity[3]. Fixed biomarker requirements may consequently delay referral and contribute to the underrepresentation of obesity-related HFpEF in clinical trials. Low natriuretic peptide concentrations remain prognostically informative and generally identify lower-risk disease than markedly elevated values, but they neither establish normal exercise hemodynamics nor explain persistent functional limitation.

## A phenotype-aware diagnostic pathway

Evaluation should begin with clinical probability rather than an isolated biomarker result. Central obesity, hypertension, type 2 diabetes, atrial fibrillation, obstructive sleep apnea, chronic kidney disease, previous congestion, and disproportionate exertional limitation should heighten suspicion. Natriuretic peptide concentrations should then be interpreted in the context of age, cardiac rhythm, renal function, diuretic use, and whether the presentation is acute or ambulatory. Although obesity-adjusted thresholds have been proposed, they are not sufficiently standardized to replace integrated clinical assessment[7].

Resting echocardiography should evaluate ventricular geometry, left atrial size, tissue Doppler velocities, pulmonary pressures, right ventricular function, and valvular disease. Validated probability scores can organize these findings. The H₂FPEF score incorporates obesity, antihypertensive medication use, atrial fibrillation, pulmonary hypertension, age, and estimated filling pressure to distinguish HFpEF from non-cardiac causes of dyspnea[8]. The Heart Failure Association Pre-test Assessment, Echocardiography and Natriuretic Peptide, Functional Testing, and Final etiology algorithm integrates functional, morphological, and biomarker domains. However, obesity-related natriuretic peptide suppression may reduce the biomarker contribution to this score[9]. Neither approach should be interpreted as a definitive binary test.

Patients with a low probability of HFpEF should be evaluated for alternative cardiac and non-cardiac causes, whereas a high probability supported by concordant structural or hemodynamic evidence may be sufficient to establish the diagnosis. Intermediate or discordant presentations require particular attention. Exercise echocardiography can reveal elevations in filling pressures that are absent at rest[10]. Cardiopulmonary exercise testing can further characterize the source of functional limitation, while invasive exercise hemodynamic testing remains the reference standard when uncertainty persists, and diagnostic confirmation would alter management. A normal BNP or NT-proBNP should not prompt indiscriminate testing, but it should not close the diagnostic pathway when the symptoms and clinical phenotype remain compelling.

## Treatment and trial implications

Recognition of obesity-related HFpEF now carries direct therapeutic implications. In EMPEROR-Preserved, empagliflozin reduced the risk of cardiovascular death or heart failure hospitalization in patients with symptomatic heart failure and an ejection fraction above 40%, with the benefit driven primarily by fewer heart failure hospitalizations[11]. STEP-HFpEF subsequently showed that semaglutide reduced weight and improved heart failure-related symptoms, physical limitations, and exercise capacity in patients with obesity-related HFpEF[12]. In SUMMIT, tirzepatide reduced the risk of cardiovascular death or worsening heart failure and improved health status[13]. These findings make diagnostic accuracy increasingly consequential, but they do not justify assigning an HFpEF diagnosis or initiating phenotype-specific treatment without objective confirmation.

Trial eligibility also warrants reconsideration. Requirements for elevated natriuretic peptide concentrations can improve diagnostic certainty and enrich trial populations for clinical events, but may underrepresent patients with obesity and lower concentrations[3]. Future trials should consider phenotype-sensitive eligibility criteria and prospectively evaluate whether treatment effects remain consistent across the natriuretic peptide spectrum.

## Conclusion

A normal BNP or NT-proBNP concentration lowers the probability of HFpEF but does not universally exclude it in patients with obesity. When symptoms and the cardiometabolic phenotype remain compelling, evaluation should proceed through integrated clinical assessment and, when appropriate, exercise-based testing. Natriuretic peptides should remain central to HFpEF diagnosis but should function as context-dependent evidence rather than an isolated diagnostic gatekeeper.

## Ethical approval

Not applicable. This editorial is based exclusively on previously published literature and did not involve human participants, patient-level data, or animal subjects.

## Consent

Not applicable. No patient information or identifiable patient data are included in this editorial.

## Data Availability

Data sharing is not applicable to this article because no datasets were generated or analyzed during its preparation.

## Research registration unique identifying number (UIN)

Not applicable. This article is an editorial and does not involve a clinical trial, systematic review registration, or prospective research registration requiring a unique identifying number.

## References

[1] Zakeri R Nabeebaccus AA. Heart failure with preserved ejection fraction (HFpEF): diagnosis and management for the general physician. Clin Med (Lond) 2026;26:100608. 10.1016/j.clinme.2026.100608 PMC13356613 42331290

[2] Gupta M Kumar D Gupta T. Optimizing the use of N-terminal pro-B-type natriuretic peptide (NT-proBNP) in the diagnosis of heart failure with preserved ejection fraction (HFpEF): a clinical pathway approach to an underdiagnosed entity. Cureus 2025;17:e99161. 10.7759/cureus.99161 PMC12796551 41536407

[3] Borlaug BA Jensen MD Kitzman DW. Obesity and heart failure with preserved ejection fraction: new insights and pathophysiological targets. Cardiovasc Res 2022;118:3434–50. 10.1093/cvr/cvac120 PMC10202444 35880317

[4] Obokata M Reddy YNV Pislaru SV. Evidence supporting the existence of a distinct obese phenotype of heart failure with preserved ejection fraction. Circulation 2017;136:6–19. 10.1161/CIRCULATIONAHA.116.026807 PMC5501170 28381470

[5] Verbrugge FH Omote K Reddy YNV. Heart failure with preserved ejection fraction in patients with normal natriuretic peptide levels is associated with increased morbidity and mortality. Eur Heart J 2022;43:1941–51. 10.1093/eurheartj/ehab911 PMC9649913 35139159

[6] Kondo T Campbell R Jhund PS. Low natriuretic peptide levels and outcomes in patients with heart failure and preserved ejection fraction. JACC Heart Fail 2024;12:1442–55. 10.1016/j.jchf.2024.04.027 38904646

[7] Taylor CJ Taylor KS Jones NR. Age-adjusted natriuretic peptide thresholds for a diagnosis of heart failure in the community: diagnostic accuracy study. ESC Heart Fail 2025;12:3552–68. 10.1002/ehf2.15383 PMC12450762 40717271

[8] Reddy YNV Carter RE Obokata M. A simple, evidence-based approach to help guide diagnosis of heart failure with preserved ejection fraction. Circulation 2018;138:861–70. 10.1161/CIRCULATIONAHA.118.034646 PMC6202181 29792299

[9] Pieske B Tschöpe C de Boer RA. How to diagnose heart failure with preserved ejection fraction: the HFA-PEFF diagnostic algorithm. Eur Heart J 2019;40:3297–317. 10.1093/eurheartj/ehz641 31504452

[10] Obokata M Kane GC Reddy YNV. Role of diastolic stress testing in the evaluation for heart failure with preserved ejection fraction: a simultaneous invasive-echocardiographic study. Circulation 2017;135:825–38. 10.1161/CIRCULATIONAHA.116.024822 PMC5330848 28039229

[11] Anker SD Butler J Filippatos G. Empagliflozin in heart failure with a preserved ejection fraction. N Engl J Med 2021;385:1451–61. 10.1056/NEJMoa2107038 34449189

[12] Kosiborod MN Abildstrøm SZ Borlaug BA. Semaglutide in patients with heart failure with preserved ejection fraction and obesity. N Engl J Med 2023;389:1069–84. 10.1056/NEJMoa2306963 37622681

[13] Packer M Zile MR Kramer CM. Tirzepatide for heart failure with preserved ejection fraction and obesity. N Engl J Med 2025;392:427–37. 10.1056/NEJMoa2410027 39555826

